# Factors Influencing Health-Related Practices Among Hispanic Parents: A Formative Study to Inform Childhood Obesity Prevention

**DOI:** 10.3390/children12070887

**Published:** 2025-07-05

**Authors:** Ana Paola Campos, Julian Robles, Katherine E. Matthes, Maihan B. Vu, Ramine C. Alexander, Rachel W. Goode

**Affiliations:** 1 Nutrition Research Institute, University of North Carolina at Chapel Hill, 500 Laureate Way, Kannapolis, NC 28081, USA; julianr6@live.unc.edu (J.R.); katherine_matthes@unc.edu (K.E.M.); raminea@unc.edu (R.C.A.); rwgoode@email.unc.edu (R.W.G.); 2 Connected Health Applications and Interventions Core, University of North Carolina at Chapel Hill, Chapel Hill, NC 27599, USA; maihan.vu@unc.edu; 3 Center for Health Promotion and Disease Prevention, University of North Carolina at Chapel Hill, Chapel Hill, NC 27599, USA; 4 Gillings School of Public Health, University of North Carolina at Chapel Hill, Chapel Hill, NC 27599, USA; 5 School of Social Work, University of North Carolina at Chapel Hill, Pittsboro St. CB #3550, Chapel Hill, NC 27599, USA

**Keywords:** parenting practices, childhood obesity prevention, formative research, Hispanic families

## Abstract

**Background:** Childhood obesity disproportionately affects Hispanic families in the U.S. Although parenting practices and interventions appear to be viable approaches to lower the risk of childhood obesity, there is limited information on which parenting practices would be relevant and culturally appropriate for Hispanic families. This study aimed to document the health-related factors that impact Hispanic parenting practices to inform evidence-based parenting interventions to improve child health outcomes and reduce the risk of childhood obesity. **Methods:** We conducted cross-sectional, formative research guided by the COREQ checklist. Hispanic parents of elementary school-aged children were recruited using purposive and snowball sampling. Eligible parents completed a brief sociodemographic survey, the Comprehensive Feeding Practices Questionnaire (CFPQ), and an individual semi-structured interview. Quantitative data were analyzed using descriptive statistics, and qualitative data were analyzed using a coding-based thematic approach. **Results:** Seventeen parents (88.2% female) participated in the study, and the majority reported Spanish as their preferred language (70.6%). According to parent-reported data, the children’s (52.9% female) mean age was 8.4 ± 1.5 years, and over half were classified as having overweight or obesity. The CFPQ analysis indicated that parents scored highest on subscales related to encouraging healthy eating and eating variety, a finding that was reinforced by interview data. Qualitative analysis identified four key themes: 1. parenting roles, routines, and strategies for promoting culturally appropriate and healthy meals; 2. beliefs of children’s health and weight; 3. beliefs on physical activity and screen time; and 4. environmental and social factors affecting access to healthy food and physical activity. **Conclusions:** Both quantitative and qualitative results emphasize that parents engage in healthy eating guidance and eating variety and are aware of the factors that impact parenting practices and their children’s health. To improve child health outcomes and reduce the risk of childhood obesity among Hispanic families, parenting interventions need to be culturally responsive and address the factors that influence parenting practices. The findings from this study highlight four key areas to prioritize when designing or adapting parenting interventions to lower the risk of childhood obesity among Hispanic families.

## 1. Introduction

Childhood obesity remains one of the most challenging and common pediatric chronic diseases worldwide, with prevalence rates rising despite decades of intervention efforts [1,2,3]. In the United States of America (U.S.), Hispanic children have the highest prevalence of obesity (26.2%), displaying a disproportionate burden compared to non-Hispanic children (Black 24.8%, White 16.6%, and Asian 9.0%) [4,5]. Childhood obesity is a complex disease influenced by the interaction of genetic, epigenetic, and environmental factors, all of which contribute to its development [6]. Children with obesity are five times more likely to remain with obesity during adulthood, particularly those with one or both parents with obesity, given their shared genetics and obesogenic environments (i.e., proximal and distal environmental factors that shape diet-related and physical activity behaviors) [6,7,8]. Furthermore, childhood obesity increases the likelihood of short- and long-term adverse health outcomes, including type 2 diabetes mellitus, cardiovascular diseases, orthopedic problems, and psychological disorders, among many others [1,9]. Despite numerous policies and interventions aimed at preventing childhood obesity, persistent high prevalence rates continue to affect Hispanic children and families in the U.S.

A growing body of literature has established that across diverse racial and ethnic groups, including among Hispanic families, parents/caregivers largely shape their children’s health-related behaviors, thus being able to prevent negative child health outcomes such as obesity [10,11,12]. Parental influence on their children’s behaviors seems to be relevant among younger children (≤12 years) rather than older counterparts who may have shifted role-modeling guidance towards peers rather than parents [13]. We know that positive parenting practices and behaviors may be efficacious components of interventions for childhood obesity prevention [11,12]. A recent systematic review provides evidence that some parenting practices, such as setting limits and establishing routines, supporting a healthy lifestyle and physical activity, and parenting feeding practices, may lower the risk of childhood obesity among Hispanic parents [12]; however, there seem to be some discrepancies, and there is no consensus on which parenting practices, behaviors, and beliefs would be relevant and culturally-appropriate for Hispanics to improve family health behaviors and child health outcomes [12,14]. Additionally, there is limited information on optimal strategies to engage and retain Hispanic parents on interventions; parenting interventions for Hispanics have faced recruitment and retention challenges, language and cultural barriers, and difficulty building trust [12,14,15].

There is, therefore, a critical need for formative research (i.e., an exploratory approach used to understand the behaviors, beliefs, and contextual factors of a target population that can inform the design and content of tailored interventions) to guide the future development of an evidence-based, culturally appropriate childhood obesity prevention intervention for Hispanic families [14]. Existing interventions often lack cultural specificity, and few studies have examined the parenting practices and beliefs that shape health-related behaviors in this population [14]. The objective of this study was to explore and document the factors influencing Hispanic parents’ health-related practices, behaviors, and beliefs to inform the development of a future parenting intervention to reduce the risk of childhood obesity.

## 2. Methods

### 2.1. Study Design

The study design was cross-sectional and collected qualitative and quantitative data to document health-related parenting practices, behaviors and beliefs, and the factors that influence these practices. Although the study included both qualitative and quantitative data collection methods, it was not designed as a mixed-methods study. The approach was exploratory and complementary rather than integrative; data sources were collected and analyzed separately and not combined to answer a single central research question. Instead, the findings were used complementarily to explore whether both approaches converged or not [16]. This research was guided by the Consolidated Criteria for Reporting Qualitative Research (COREQ) checklist, including the three domains: (1) research team positionality statement and reflexivity, (2) study design, and (3) report on analysis and findings [17]. The methodology was grounded in a community-engaged research approach to ensure that the participants were not just sources of data but also active contributors to the research process and that our community partners were engaged [18]. Recruitment, consent, enrollment, and data collection were conducted between June and December 2024.

### 2.2. Participants’ Recruitment

In June 2024, recruitment efforts began by approaching non-profit Hispanic-serving organizations that offer education programs and health-related services and University-based centers in North Carolina, U.S. After presenting the study protocol to directives from those organizations and gaining approval, the research team distributed recruitment flyers with a QR code for screening purposes within those organizations. Potential participants scanned the QR code in the recruitment flyers and completed the screening questionnaire and provided their contact information. Using a combination of purposive and snowball sampling strategies [19], we aimed to enroll 16 to 20 parents who identified as Hispanic and had elementary school-aged children, 8–10 of whom had children with overweight/obesity and 8–10 of whom had children with no overweight/obesity. Interviewing these two distinct groups will aid in understanding, in a future analysis, if parenting practices differed or not among those with and without childhood overweight/obesity. Eligibility criteria to participate in the study included the following: (1) parent/caregiver aged 18 years or older, (2) self-identified as Hispanic/Latino, (3) able to speak in English, and (4) had a child or was responsible for a child attending elementary school at the time of the interview. Eligible participants were contacted by the research team using the information they provided in the screening questionnaire to facilitate further information and assess enrollment to the study.

During the recruitment period, the third eligibility criterion, ability to speak English, was identified as a significant barrier to participation by the research team, community members, and leaders, which limited the study’s ability to capture the voices of the most vulnerable members of the target population. All research team members considered modifying the eligibility criteria to allow parents who spoke Spanish only, in addition to English-speaking Hispanic parents, to participate in the study; after consensus, an addendum was submitted to the Institutional Review Board (IRB) to include Spanish surveys, interviews, and recruitment material, and the IRB considered the protocol to remain exempt. Subsequently, Spanish recruitment flyers, digital and hard copies, were distributed mostly through a non-profit Hispanic-serving organization.

Eligible participants were asked to read and sign a written informed consent prior to data collection, and upon study completion, the participants were compensated with a USD 120 digital or physical gift card.

### 2.3. Data Collection

#### 2.3.1. Quantitative Data

The participants received a survey link via their preferred method of communication, either email or text message. Quantitative data were collected using a brief socio-demographic survey, which included self-reporting the child’s weight and height at the time of the survey, birthdate, sex at birth, as well as a question asking the parents whether they perceived their child to have overweight or obesity. We also collected data using the 34-item, 5-subscale Comprehensive Feeding Practices Questionnaire (CFPQ), a measure of parenting feeding practices, which has been validated among the Hispanic population and published [20]. For the Spanish version of the CFPQ, we used formerly translated versions that were applied in previous studies [21,22,23]. The 34 items of the CFPQ are rated on a 5-point Likert scale, with response formats varying depending on the item type: frequency-based items (1 = never to 5 = always) and agreement-based items (1 = disagree to 5 = agree). The 5 subscale scores are calculated by computing the mean of the items within each subscale. The 5 subscales include the following: monitoring (4 items) assesses how parents keep track of their children’s consumption of various food types such as sweets, sugary drinks, and high-fat foods; restriction (7 items) assesses portion control, particularly as it relates to controlling the child’s body weight; promotion of overconsumption (8 items) assesses parents’ use of food to soothe their children’s emotions or reward good behaviors; healthy eating guidance (12 items) assesses how parents modeled healthy eating and the availability of healthy foods in the house; and healthy eating variety (3 items) assesses how parents speak about food and variety and encourage their child to consume healthy foods. Higher scores indicate greater parental use of that particular feeding practice [20].

#### 2.3.2. Qualitative Data

After completing the survey described above, the participants were contacted via their preferred method of communication, either by telephone and/or email, to schedule an individual interview according to their most convenient day, time, and format (telephone or in-person). Semi-structured individual interviews were used to collect qualitative data and were analyzed using thematic methods to identify, organize, and interpret patterns in the data [18]. The interviews were conducted utilizing a semi-structured guide developed by the research team and informed by a recent systematic literature review on parenting practices among Hispanic families to prevent childhood obesity [12]. The interview guide included open-ended questions that led the conversation around health-related parenting practices and behaviors (see Supplemental Table S1). All interviews were facilitated by researchers with qualitative methods training. Spanish interviews were conducted by native bilingual (English and Spanish) researchers.

### 2.4. Data Processing and Analyses

#### 2.4.1. Quantitative Analysis

STATA (v. SE 16.1) was used for all quantitative estimations. Descriptive statistics were used to characterize the sample. Cronbach’s alpha coefficients were estimated to assess the internal consistency of the 5 CFPQ subscales. Means and standard deviations (SDs) were estimated for each individual item and each of the subscales of the CFPQ. We estimated the children’s CDC percentiles using the sex-specific BMI for age to classify children as having underweight, healthy weight, overweight, or obesity [24].

#### 2.4.2. Qualitative Analysis

Interviews were digitally recorded and transcribed verbatim using artificial intelligence (AI) transcription services: English interviews were transcribed using TEMI [25], and Spanish interviews were transcribed using SONIX [26], both of which are AI powered. For the qualitative data analysis, a thematic content analysis approach was used. Each transcript was reviewed for quality and accuracy and then uploaded to DEDOOSE (v. 9.0.107), a qualitative software package. We developed a codebook based on the interview guide questions and the recurrent themes found in the transcripts. In addition, two independent coders experienced in the analysis of qualitative data reviewed the interviews and independently coded the transcripts. We initially pilot tested by independently coding two English and two Spanish interview transcripts, which led to fine-tuning concept definitions and revising coding decisions [26]. This process continued until replicability occurred across coders. Standard consensus coding guidelines were followed, in which discrepancies between coders were identified, discussed, and reconciled by the research team [27]. Once consensus was reached, and all transcripts were fully coded, the finalized codes were reviewed and grouped into broader categories. Through team discussions, these categories were further developed into themes, guided by patterns across transcripts and the study’s research objectives.

## 3. Results

### 3.1. Descriptive Results

Overall, 17 Hispanic parents (88.2% female) participated in the study. Of these, 47.1% completed some high school or less, and the majority (76.5%) were married or co-habiting with their partner. The children were 52.9% female, with a mean age of 8.4 ± 1.5 years. Using the sex-specific BMI-for-age percentiles classification [24], 17.6% (n = 3) of children were classified as having underweight, 29.4% (n = 5) as having a healthy weight, 35.3% (n = 6) as having overweight, and 17.6% (n = 3) as having obesity (Table 1). Of the nine children classified as having overweight or obesity based on sex-specific BMI-for-age percentiles, six parents (66.7%) did not perceive their child as having overweight or obesity. Parental perception was assessed via a survey question asking whether they believed their child had overweight or obesity. Five participants (29.4%) completed the surveys in English and the interviews by telephone in English. Three participants (17.6%) completed the surveys in English and preferred to complete the interviews in Spanish by telephone. Nine participants (52.9%) completed the surveys in Spanish and the interviews in person in Spanish; a total of 10 Spanish in-person interviews were conducted, but one participant had incomplete child anthropometric data. After several unsuccessful attempts to contact her, the team was unable to use her data. In-person interviews were conducted individually in the classrooms of the non-profit Hispanic-serving organization.

### 3.2. Quantitative Results

The results from the CFPQ are summarized in Table 2. Four of the five CFPQ subscales demonstrated good or excellent internal consistency (α > 0.80). Overall, the subscales with the highest mean scores were healthy eating variety (4.71 ± 0.41) and healthy eating guidance (4.13 ± 0.67). Parents scored the lowest in the promotion-of-overconsumption subscale (2.36 ± 0.67), which also demonstrated the lowest internal consistency (α = 0.64).

### 3.3. Qualitative Results

On average, the interviews lasted 38 min (range: 20–62 min). Four key themes were identified, each illustrating different aspects that shape health-related parenting practices, behaviors, and beliefs: 1. planning culturally appropriate and nutritious meals and establishing family routines; 2. parent beliefs on children’s health and weight; 3. parent beliefs on physical activity and screen time; and 4. access to community resources for physical activities and nutritious food. These findings and additional illustrative quotes are summarized in Table 3.

**Theme 1. Parenting Roles, Routines, and Strategies for Promoting Culturally Appropriate and Healthy Meals.**

The participants shared their experiences and roles as parents and/or caregivers, including routines and schedules for meals on school days and weekends. Meal planning and preparation roles were often done by one parent; however, it varied across households, with some reporting that one parent had all these roles while others shared these roles with their partner or that the entire family was involved in the process, as described by two participants in the following quotes:

*Uh, really depends. Both my wife and I work, so whoever has time [to buy and prepare meals].*

[P 118]

*My husband buys the food, I prepare it. We buy fruit as well as vegetables.*

[P 121S]

Participants described the types of meals they prepared for their families. They noted a few key aspects of these meals. This included preparing meals that were culturally relevant and reflective of their traditional food practices, preparing homemade foods, including vegetables in creative manners, and using different cooking methods and devices such as crockpots.

*Uh, we eat, uh, always, bringing Cuban food. We also eat a lot of vegetables, we eat a lot of fruits. Rice should never be missing from the table. The grains could, well, be beans in any of its, of its types as such.*

[P 114S]

*Chicken Molito [spicy Mexican stew]. Eh spaghetti, and now, as my husband is from Guerrero [a southwestern state in Mexico], well, I prepare Pozole [another traditional Mexican stew].*

[P 125S]

Most participants talked about their daily schedules in similar ways. Their weekday routines centered around getting their child ready for school, going to school, going to work, and then coming back home to prepare dinner. As the participants shared their schedules and routines, they highlighted how they handled different meals during their typical weekdays. This included breakfast eaten at home with quick and easy items; lunches eaten at school, which varied depending on the child’s like of the school food options, children feeling comfortable with the homemade lunches, or parents approving of school lunches; and dinners eaten together at home with the family, which were often described as a good time to talk about how the day went.

*Breakfast is a little different because my kids eat breakfast before they go to school. So, when they go to school it is something fast to prepare like a quesadilla [tortilla with cheese]. If I have time I make pancakes. If not and quick little things like that, sometimes they would be, you know, sometimes I make them a slice of bread with beans.*

[P 126S]

*We see it well at home, but at school, well, what I do know is that eat, they don’t eat healthy at school. So, I would be lying to you that my child doesn’t want me to send him [lunch] either because he says, no, how embarrassing that I’m going to carry my, my, my, my, this [cultural foods]”. My child, “no, my classmates don’t eat like that”. Then sometimes, as the children grow up, they feel shame. So neither, right? “They are going to bully me because I bring this [cultural foods], I don’t bring that or because I don’t, then no. I’ll eat my school [lunch]”. But I know they eat pizza, I know. They eat other things that are not healthy”.*

[P 123S]

The participants talked about different ways their weekend schedules and routines changed from their weekdays. They brought up factors that impacted their weekend schedules and routines, which included more time to prepare meals and to eat together, more opportunities to eat outside of the home, more activities to do, and more time to sleep in or stay up late.

*Uh, well, on weekends, they usually wake up at 10 in the morning, almost 11. I don’t, I don’t get them up that early because they are days that are and they rest.*

[P 122S]

The participants indicated there was a familiarity and comfort with their schedules and routines and noted that their children were also aware of these weekday and weekend schedules.

*So we sit down at the table, have breakfast. I drive him to school and then he comes back around 2 in the afternoon. Around 3 in the afternoon. When he returns, he always has a snack when he gets home. We talked about the day and how he did in school. He has a routine here at home that corresponds to him. Pick up the trash, organize the room in case, well, if he left a mess. Clean the surroundings outside. Blow away the grass. Anything like that.*

[P 114S]

The participants shared different examples of both healthy and unhealthy meals and talked about healthy foods having certain characteristics, including cooking with little oil or fat; being organic or natural; providing sustainment, fuel, and energy; and being prepared in the home.

*It [healthy foods] should have vegetables, protein and a little bit of carbohydrate. Because I have learned that with one’s Hispanic roots one thinks that carbohydrate is going to fill me up. And I’ve learned that it’s not the carbohydrate. It is the protein that will sustain me.*

[P 129]

Additionally, the participants talked about unhealthy foods having specific characteristics, including being cooked or fried with oil, processed/not natural, containing fats, not a source of energy, not homemade/prepared outside of the home.

*[When I eat unhealthy foods] well, I feel tired. I feel no energy. A lot of, I just want to be asleep and I don’t, I don’t have any energy to do anything.*

[P 117]

When discussing healthy and unhealthy meals, the participants highlighted different challenges that made it difficult for their child to eat healthy, and in some cases, the parents shared their own barriers to healthy eating. Some of the barriers included the following: the child is a picky eater, the child does not like the taste, smell, or texture of certain foods (vegetables), the child prefers school foods (which parents consider unhealthy), the child wants to eat what their peers eat due to concerns about eating cultural foods that are different, and there are higher costs for healthier foods versus cheaper options for processed food.

*Because he is, my little kid is very picky about healthy foods. I mean, every child of that age I think is like that. He likes the food of the, like his, he loves French fries.*

[P 122S]

The participants shared their strategies and ideas for helping their child to eat healthy which included creatively incorporating vegetables into foods children like, preparing foods at home, encouraging children to participate in meal preparations, modeling and seeing parents eat healthy, providing verbal encouragement to try new/healthy food, discussing the impact of food choices on future health, discussing the need to limit food amounts, discussing the connections of healthy eating to physical health and strength, and discussing the connections of healthy eating to avoid going to the doctor.

*And, and sometimes I’d be like, okay, if you eat it, you’ll be strong. And then my daughter is, and my daughter is the kind of person, she said she wants her hair long. So, I said, okay, if you eat it [healthy food], your hair’s gonna grow.*

[P 112]

The parents shared how they handle sweets or desserts in their homes. Strategies included limiting the purchase of sweets and desserts; limiting the amount, portion, or quantity served; limiting sweets to certain times of the day or to certain days or occasions; offering sweets only as a treat or reward; and diluting sweet drinks or juices with water.

*But then again, I buy her a pack of Oreo cookies every once in a while and she has to be eating them a little bit at a time, but she doesn’t sit down to finish them, right? … I tell her do not [eat] much. You can only eat two or three and then some more [later].*

[P 127S]

**Theme 2.Parents’ Beliefs on Children’s Health and Weight.**

The participants discussed their views on what it means for their child to be healthy and shared their thoughts on overweight and underweight children and talked about their views on their own child’s weight. The parents commented on the factors that may impact their child’s health and bodyweight in negative ways; some included the child being a picky eater, not wanting to eat healthy, wanting to eat sweets and candy, and the child feeling that they are “fat”.

*When sometimes she looks in the mirror and she’s like, she’s like, mommy, I look I’m fat, look in my belly. And I’m like, no, you’re not.*

[P 112]

The participants shared what it means for their child to be healthy and related to the health goals they had for their child. They discussed different factors that help them identify their child as healthy, including that a healthy child eats fruit and vegetables, makes good food choices, limits the amount of food eaten, has energy, is physically active, takes care of their mental health, and is not sick.

*Healthy would be eat a balanced diet and have some kind of exercise in his day and also just take care of his mental health. ‘cause you can’t do, you can’t eat good or go outside and work out if you are not mentally in a good place to do those things.*

[P 123]

The parents also described the characteristics they think would apply to children with overweight, including that they eat more snacks and sweets, eat less fruits and vegetables, are not physically active or agile, are not healthy, may have an eating disorder, and spend more time on screens and electronic devices; the parents note that this may have a genetic component.

*Well, what I think is that maybe they eat too many snacks. Too many. Ah, yes, well, snacks or things that are fattening, I don’t know, donuts, bread, cookies, Sabritas [potato chips].*

[P 125S]

The parents identified positive characteristics of children with healthy weight and the negative characteristics of children with underweight, including that a child with healthy weight has more energy and a child with underweight has a lower immunity, is more likely to get sick, and is not getting enough nutrition for growth.

*Because if they are underweight, there is a little problem and, as I said, we have to give them more food so that the child gains weight.*

[P 119S]

The participants talked about their views of their child’s weight, which varied across the sample from healthy weight, overweight, or underweight.

*When she had her medical checkup, I was told that she was fine in weight, that she was fine in height. In fact, she was thin before, but they told me she was fine, that there was no problem. Recently she has gained a little weight. Yes, I feel that she is eating a little more, but she is in a very normal range. So no, it’s not that she is overweight.*

[P 125S]

**Theme 3. Parents’ Beliefs on Physical Activity and Screen time.**

The participants discussed their views on the physical activity goals they have for their child and the types of physical activities that their child does. Additionally, they shared their involvement with their child’s physical activities and outlined their experiences with their child and screen time. The participants were asked about any physical activity goals they had for their child. They talked about a few different things that were included in these goals, such as wanting their child to have a good balance of healthy eating and exercising, be strong and fit, play out and be outdoors, run around and be athletic, and participate in activities that they did when they were younger.

*For me, he can be outside playing for two or three hours. To me, that’s fine, because he’s playing something in his mind better.*

[P 121S]

Additionally, the participants talked about the importance of their child being physically active. As a participant explained, physical activity was important to help their child grow and have strength:

*Because it is always good to exercise, you have to stimulate the muscle because if you don’t stimulate it, it atrophies and then you have to do things.*

[P 114S]

The participants noted that their child participated in different sports and physical activities such as biking, dancing, fishing, gardening, playing with pets, playdates at the park, running, and walking. Additionally, they mentioned that their child participated in organized activities at school or on the weekends such as soccer or karate:

*So again, it depends on if it’s a Saturday or Sunday. Um, because, you know, Saturday there’s Ninja. Right now she has a ninja challenge. Okay. Class that’s at 9, 9:15.*

[P 118]

It was common for the participants to report that their child participated in more leisure and less structured activities:

*They play inside and they play outside. But when they go outside, they’re on the bike, on the swings, things like that.*

[P 128S]

The parents shared different ways in which they were involved with their child, such as biking, dancing, going to the park, running errands, playing games, running, walking, or practicing sports together.

*Well, sometimes I tell her, “Let’s go play soccer”. If there are four of us. Because I tell my husband, go play with them.*

[P 115S]

The participants shared their experiences about their child and the use of devices, including cell phones, computers, laptops, and tablets. Activities commonly done on devices were watching shows, movies, or videos; playing video games; being on video-based social media platforms; drawing pictures while looking at the phone; and taking photos. The parents also shared their strategies for limiting screen time, including setting time limits on devices, limiting device use until chores were completed, encouraging the child to do another activity, turning off or taking away devices, and not making devices available to their child.

*So I try to, um, make them be more active and try to, okay, you know what, it’s time for you to turn that off and go do something, play around, play with something, but just leave that electronics system because I got, um, I don’t know how some kids nowadays they’d be like, they can’t be without no phone, no tablet, none of that stuff. And I’m like, I grew up with no phone, no tablet, nothing.*

[P 112]

In contrast, some explained they did not have set limits or rules for their child about screen time if it was appropriate, supervised, or not an obsessive behavior. According to a participant:

*Screen time is unlimited as long as it’s appropriate.*

[P 113]

The participants highlighted certain situations when they allowed their child to use devices more frequently, which included the weekends, after school or during a break, and when children were at adult-only functions after church social activities.

*She has an iPad, but she is only allowed to use it twice a week and she uses it on Fridays and Saturdays, or sometimes Saturday and Sunday and not all day. Just one hour.*

[P 126S]

**Theme 4. Environmental and Social Factors Influencing Children’s Health.**

The participants were asked about the things that positively and negatively impact children’s health. The participants described a variety of social and environmental factors negatively impacting their children’s health. Factors such as the child lacks friends or family for playmates, the child dislikes going outside, the child is served unhealthy lunches at school, the participants are not on top of their child’s diet, the parents do not have the flexible schedules or help with transportation for physical activities, a lack of local health clinics, a lack of farmers’ markets, and the high cost of healthy foods and physical activity opportunities.

*And sometimes I realize that they are giving free classes of this and that but sometimes because of the work as a Hispanic you leave work late, so you don’t have time to take your children to classes or sometimes they have programs in the schools and you can’t leave them because you don’t have anyone to pick them up.*

[P 129S]

*Ah, well, at Walmart I go there, right, to stores like this. But when one goes to the farmers' market like that, well yes [produce is less expensive], there is one where Americans go, it’s about 12 min from home.*

[P 115S]

Conversely, the participants commented on the factors that may impact their child’s health and bodyweight in positive ways at the individual, social, and environmental levels. Factors on the child’s individual level include the child having good eating habits and that the child is aware of the need to be active.

*I think she is aware that she has to do some kind of physical activity.*

[P 126S]

The participants also described a variety of social and environmental factors negatively impacting their children’s health; factors such as having a parent or caretaker get them involved in being active and eating healthy, parents or caretakers that offer verbal encouragement, preparing meals in creative and healthy ways, the availability of neighbors and family as playmates, availability of local parks, fresh fruits and vegetables, outdoor spaces, and community centers.

*Well. The only thing, that, [she] comes to the Hispanic center here. She does her homework, has a little fun with the kids.*

[P 127S]

## 4. Discussion

This study aimed to document the factors that influence Hispanic parents’ practices, behaviors, and beliefs to inform evidence-based interventions to improve child health outcomes and reduce the risk of childhood obesity among Hispanic families. Four main themes summarize what the participants shared during the interviews. Qualitative and quantitative data highlighted the factors that influenced parents’ behaviors and indicated that parents practiced healthy eating guidance and healthy eating variety, in addition to being aware of the impact of a healthier lifestyle on their children’s health yet not being able to identify their children’s weight status. The parents were able to identify several barriers and facilitators to healthy lifestyles while also being able to share some strategies to overcome barriers to healthful parenting practices.

Our findings suggest that the parents in our sample were aware of their key role in facilitating and supporting healthy lifestyles for their children, as well as the benefits of preparing meals at home. The parents emphasized cultural food tradition and accommodating different family members’ food preferences. Meal preparation routines varied depending on the day of the week, with simplified meals during weekdays. In general, daily routines for parents and children varied depending on several factors, such as working and school schedules on weekdays, and familial leisure preferences during the weekend. These findings align with prior research indicating that Hispanic mothers often prepare culturally traditional food and multiple dishes to satisfy varied tastes within the household [28,29,30]. When designing interventions, including components such as culturally relevant meal practices, flexible routines, cooking lessons that include traditional ingredients, and practical strategies for managing family dynamics can help empower parents to lead healthier lifestyles that impact positively their children’s health [31].

Since most of our participants were women, these findings continue to demonstrate that Hispanic mothers and female caregivers play a significant role in their family’s daily and feeding behaviors [32]. Traditionally, Hispanic mothers are responsible for the health of their family through nutrition, which allows them to determine which foods are available in the household and what meals are prepared throughout the day [32]. Considering that many of the mothers or female caretakers in our sample buy the household groceries and prepare traditional cultural meals for their families, mothers or female caretakers play key roles in influencing parent practices to prevent childhood obesity. However, evidence supports the idea that other family members, including children and fathers, need to be included in the process of meal preparation and feeding practices to have a positive impact on the entire family and improve the household food environment [33].

The parents were able to clearly differentiate the benefits of children’s healthy bodyweight, and the risks associated with underweight, overweight, and obesity. Despite this awareness, not all parents of children classified as having overweight or obesity recognized their child’s weight status accurately, meaning that they underestimated their children’s overweight/obesity. This finding is consistent with the previous literature indicating that Hispanic parents, particularly those of Mexican origin, tend to underestimate their child’s body weight, not being able to identify their child as having overweight or obesity, and associating higher child body weight with health and well-being [34]. These insights highlight the importance of culturally informed intervention strategies that go beyond reinforcing nutritional knowledge to also address culturally rooted perceptions of healthy weight. Such strategies are essential to support more accurate parental recognition of weight-related issues and to foster meaningful engagement in early prevention efforts. Furthermore, additional research is needed to identify culturally appropriate communication strategies targeted at Hispanic parents that avoid potentially judgmental language, allowing for variability in body size preferences and focusing on components of a healthy lifestyle rather than prescribing an ideal body weight for children [34,35].

Both the qualitative (interviews) and quantitative findings (CFPQ) showed that the parents in our study could discern between healthful and unhealthful foods. The parents reported using positive feeding practices such as healthy eating guidance, encouraging dietary variety, and introducing new foods. These results are consistent with previous studies, where parents identified barriers to healthy eating but often found creative strategies to overcome them [31]. For instance, in our study, during interviews, some participants shared strategies for incorporating vegetables into traditional cultural foods that children appreciate, such as stews or tacos, to increase vegetable intake. Quantitative data supported these qualitative findings. Four of the five CFPQ subscales (monitoring, restriction, healthy eating guidance, and healthy eating variety) demonstrated good internal consistency and high scores, suggesting that the items within each subscale reliably measured the same underlying concept and that parents use those positive feeding practices, correspondingly. In contrast, the promotion-of-overconsumption subscale showed low internal consistency. Given the small sample size, this finding should be interpreted with caution and may warrant further evaluation in larger samples. Nonetheless, it is worth noting that the four subscales with good internal consistency were closely aligned with key themes from the qualitative interviews. This convergence across qualitative and quantitative analysis strengthens the overall credibility of the study’s findings.

In agreement with previous research, the parents expressed concern about the nutritional quality of school-provided lunches, with several reporting that they chose, or their children often asked, to prepare homemade lunches as a healthier alternative [36]. However, some participants shared that their children requested not to include traditional cultural dishes, although the children like these foods and eat them at home, because they felt embarrassed. This experience has been described as lunch shaming, a concept in which children feel stigmatized or ashamed for culturally specific foods that deviate from dominant food norms [37]. Improvements in school-provided lunches are needed in terms of healthier and more culturally diverse foods [37,38]. Addressing lunch shaming requires a multidimensional approach that promotes the acceptance of culturally diverse foods and fosters inclusive school environments to create supportive spaces where children feel affirmed in their cultural identities. By engaging families, educators, and policymakers in these efforts, schools can play a critical role in reducing food-related stigma and supporting the psychosocial and nutritional well-being of all students [37].

The parents identified physical activity as an important component of a healthy lifestyle, which has been documented [12,39]. Most parents perceived their children as being physically active during the school week and reported that, in addition to school-based activity, their children regularly engaged in unstructured leisure activities at home with family members or friends (e.g., biking, dancing, playing in the yard, or walking to the park). Participation in structured physical activities was mentioned less frequently and occurred primarily through school programs, such as soccer, basketball, or athletics. The parents expressed an intention to enroll their children in extracurricular physical activities but identified significant barriers, including the high cost of such programs and limited parental availability to support participation. These findings are consistent with previous research highlighting time constraints as common obstacles to engaging in structured physical activity [39]. Developing low-cost, flexible strategies to incorporate fun physical activities that can be integrated into children’s routines after school and on weekends would be beneficial among Hispanic parents and their children [39].

Most parents reported having established household rules to manage their children’s screen time, while some acknowledged that they did not actively monitor or control it. Among those with rules, various strategies were described, including encouraging alternative activities such as outdoor play and negotiating screen use based on the completion of homework or other responsibilities. Despite these efforts, the parents widely recognized that screen use, while often a convenient method to keep children occupied, poses health risks, particularly due to its association with sedentary behavior and the tendency to eat while using screens. These concerns coincide with existing evidence linking excessive screen time and screen media exposure to increased risk of childhood obesity [12,40]. As such, intervention strategies should include components to monitor screen time while promoting engagement in active and non-screen-based alternatives, both during weekdays and weekends.

The parents identified a range of multilevel individual, social, and environmental factors that influence their children’s health and body weight. At the individual level, the parents noted challenges such as children’s preferences for sweets, picky eating habits, and negative self-perceptions related to body image, which may contribute to unhealthy eating behaviors and emotional distress [41]. These findings align with previous research suggesting that children’s self-perception and dietary preferences or dislikes are critical factors in shaping health-related behaviors [42]. At the social and environmental levels, the parents described significant barriers, including limited time and flexibility due to work schedules, lack of transportation, insufficient local resources (e.g., health clinics and farmers’ markets), and inadequate access to affordable healthy foods. These barriers were often compounded by structural inequities and limited social support, which is commonly reported among Hispanic women [43]. Despite these challenges, the parents also identified several protective factors, including their own role in modeling and encouraging healthy behaviors, which was also identified in the CFPQ analysis, and the benefit of local community resources such as parks and community centers such as ‘*El Puente Hispano*’. These insights highlight the importance of designing interventions that are not only culturally tailored but also responsive to the broader structural and environmental contexts in which Hispanic families live. Multilevel interventions that address both the barriers and facilitators at different levels of influence may be more effective in promoting sustained behavior change and improving child health outcomes [44].

We identified several limitations in this study. First, we relied on self-reported data for the child’s weight and height, which introduces the possibility of reporting bias and may affect the accuracy of weight status classification for children. Second, interviewer bias may have influenced how questions were asked or how responses were interpreted, particularly given that some of the interviewers shared cultural backgrounds with the participants. Additionally, the sample size was small and non-random, limiting generalizability to the broader Hispanic population. The study also may have disproportionately captured the perspectives of more engaged or health-conscious parents, especially given the use of purposive and snowball sampling. Finally, the Spanish transcripts from the interviews were translated to English for analysis by AI and revised for accuracy by native Hispanic researchers, which could have led to some loss of meaning, nuance, or culturally specific expressions during the translation process.

## 5. Conclusions

This formative research contributes to a growing body of evidence emphasizing the need for culturally grounded interventions to lower the risk of childhood obesity among Hispanic families in the U.S. [12,14,15,28,29]. We identified factors influencing parenting practices that are both relevant and potentially modifiable behaviors within the context of Hispanic cultural values and norms. The findings suggest that while many parents demonstrate knowledge of healthy eating and express concern for their children’s health and well-being, structural barriers, cultural norms, and competing demands often limit the implementation and sustainability of health-promoting behaviors. Components for such interventions should prioritize planning culturally appropriate meals that are nutritious and establishing family routines, acknowledging beliefs about children’s weight and health, prioritizing physical activity over screen time, and ensuring access to nearby community resources that support physical activity and nutritious food. This may allow for engaging parents in meaningful ways to elicit parent–child behavior change that acknowledges Hispanic cultures and values. Moreover, programs should also address several multilevel influences such as school food environments and socioeconomic constraints.

Future research is needed to build upon these insights to develop or adapt and pilot test culturally tailored parenting interventions that leverage community strengths and empower Hispanic families to support their children’s short- and long-term health. Such efforts are essential to advancing health equity and ensuring that obesity prevention strategies are inclusive, relevant, and sustainable.

## Figures and Tables

**Table 1:** Descriptive statistics. Mean (SD; range) or % (n).

Characteristics	Total Sample (N = 17)
CHILD	
Age (years)	8.4 (SD 1.5; range 6–11)
Sex at birth	
Female	52.9% (9)
Male	47.0% (8)
Percentiles classification using sex-specific BMI for age	
Underweight (<5th)	17.6% (3)
Healthy weight (5th to <85th)	29.4% (5)
Overweight (85th to <95th)	35.3% (6)
Obesity (≥95th)	17.6% (3)
CAREGIVER	
Gender	
Female	88.2% (15)
Male	11.8% (2)
Marital status	
Divorced or separated	5.9% (1)
Single	17.6% (3)
Married or co-habiting with partner	76.5% (13)
Level of education	
Some high school or less	47.1% (8)
Highschool/GED	23.5% (4)
Some college or more	29.4% (5)
Spanish as preferred language	70.6% (12)

**Table 2:** CFPQ mean and standard deviation (SD) of the 5 dimensions and 34 corresponding items.

Dimension and Corresponding Items	Sample (N = 17)
Monitoring (α = 0.90) 4 items	3.66 (0.92)
How much do you keep track of the snack food (potato chips, Doritos, cheese puffs) that your child eats?	3.53 (0.80)
How much do you keep track of the sweets (candy, ice cream, cake, pies, pastries) that your child eats?	3.71 (1.05)
How much do you keep track of the sugary drinks (soda/pop, others) this child drinks?	3.82 (0.29)
How much do you keep track of the high-fat foods that your child eats?	3.59 (0.27)
Restriction (α = 0.84) 7 items	3.06 (0.98)
I restrict the foods my child eats that might make him/her fat.	3.29 (1.36)
There are certain foods my child shouldn’t eat because they will make him/her fat.	3.24 (1.30)
I give my child small helpings at meals to control his/her weight.	3.12 (1.27)
I encourage my child to eat less so she/he won’t get fat.	3.12 (1.45)
If my child eats more than usual at one meal, I try to restrict his/her eating at the next meal.	2.94 (1.39)
I don’t allow my child to eat between meals because I don’t want him/her to get fat.	2.47 (1.37)
I have to be sure that my child does not eat too much of his/her favorite foods.	3.24 (1.48)
Promotion of Overconsumption (α = 0.64) 8 items	2.36 (0.67)
Do you give this child something to eat/drink if she/he is bored even if you think she/he is not hungry?	1.88 (1.11)
Do you give this child something to eat/drink if she/he is upset even if you think she/he is not hungry?	1.35 (0.61)
When this child gets fussy, is giving him/her something to eat or drink the first thing you do?	1.59 (0.87)
I offer my child his/her favorite foods in exchange for good behavior.	2.76 (1.60)
If my child eats only a small helping, I try to get him/her to eat more.	3.24 (1.56)
If this child does not like what is being served, do you make something else?	2.29 (1.16)
I offer sweets (candy, ice cream, cakes, pies) to my child as a reward for good behavior.	2.53 (1.46)
My child should always eat all the food on his/her plate.	3.24 (1.30)
Healthy Eating Guidance (α = 0.86) 12 items	4.13 (0.67)
I show my child how much I enjoy eating healthy foods.	4.35 (0.93)
I try to show enthusiasm about eating healthy foods.	4.65 (0.86)
I involve my child in planning family meals.	3.76 (1.39)
I model healthy eating for my child by eating healthy foods myself.	4.18 (1.13)
A variety of healthy foods are available to my child at each meal served at home.	3.94 (1.25)
I encourage my child to participate in grocery shopping.	4.35 (0.79)
I discuss with my child the nutritional value of foods.	4.35 (1.06)
I try to eat healthy foods in front of my child, even if they are not my favorite.	4.35 (0.93)
I encourage my child to try a variety of foods.	4.47 (0.72)
Most of the food I keep in the house is healthy.	4.00 (0.79)
Do you encourage this child to eat healthy foods before unhealthy ones?	3.88 (0.99)
I allow my child to help prepare family meals.	3.13 (1.67)
Healthy Eating Variety (α = 0.83) 3 items	4.71 (0.41)
I tell my child that healthy food tastes good.	4.71 (0.47)
I discuss with my child why it’s important to eat healthy foods.	4.71 (0.47)
I encourage my child to try new foods.	4.71 (0.47)

**Table 3:** Themes with illustrative quotes.

Theme(s)	Illustrative Quote(s)
Theme 1: Parenting Roles, Routines, and Strategies for Promoting Culturally Appropriate and Healthy Meals	*“In my house? I buy them and I prepare them”.* [P 128S]
	*Well, I make Peruvian food for my child, so I make him rice with chicken or noodles, red tallarines [noodles in a red sauce], his Carapulcra [pork stew], his, uh, Patasca [hominy soup].* [P 123S]
	*“We do, we do sit, um, down all together as a family when it’s, um, dinner time. And I try to make them, um, you know, spend time with us. ‘Cause during the day we’re at work and then they’re, um, at daycare. So we barely have time together”.* [P 112]
	*“Well, on weekends, from time to time. Sometimes we go out to a restaurant and there, well, wherever they (the children) like to go, right?”* [P 123S]
	*“Like if you make a pozole [hominy stew]. It already carries [contains] a lot of fat as well”.* [P 127S]
	*“For example, in other homes, uh, if their children don’t want to eat what parents prepare, they want chicken nuggets. And parents buy that. Why? Because it’s the easiest thing you can give them and sometimes because they are cheaper”.* [P 128S]
	*“Well, it [healthy diet] means a lot because he is a strong, healthy child, and if he eats well, he should be, as they say, with good defenses [resistance to sickness]. That he is well”.* [P 119S]
Theme 2: Parents’ Beliefs on Children’s Health and Weight	*“Ah, because the youngest child is very hyperactive. As long as he is moving around, he moves around, that is I see him moving around as he always knows how to move around. Well, I know that everything is going well”.* [P 122S]
	*“And with being overweight, well, I have my other girl who is a little bit like that too, and I try to support [alleviate] the anxiety that she gets from eating and eating. So it’s another way for her to eat her normal food and then, if she wants to eat, she wants to, she feels the need, keep lots of fruit so that she eats more fruit instead of eating cookies, churros, sodas, anything else”.* [P 122S]
	*“[A child is not healthy when] not doing much exercise. … I mean, one, they’re going to be tired and they’re not interested in playing outside. That’s great. Or doing any other activities”.* [P 120]
	*“Underweight? That’s worrisome, because if he eats and eats, he doesn’t gain weight. This is not normal. He has to have something [disease]”.* [P 121S]
	*“As in weight, I can consider him. Okay. Not either overweight or underweight. Uh, I’ll say in the middle”.* [P 120]
	*“I feel she is a little over, over overweight. … And I’m trying to work with her, uh, because I don’t like to see her very overweight because, uh, I don’t think it’s that healthy. But I, I try to more modify her about eat some this healthy food”.* [P 117]
	*“He’s always been a thin, a thin kid. Um, so weight has never been a problem for him. But even like I can see, I, I don’t have an ideal fit for him. It’s not something that crosses my mind. It’s not like, uh, oh I want him to be skinny or I want him to be chunky. Like, that’s not something that crosses my mind at all”.* [P 113]
	*“Well, the unhealthy [foods]. Well. These are the candy, chewing gum or maruchan [cup ramen]. Well, sometimes they do make them, because sometimes, if I’m not careful, they don’t prefer to eat the food [regular meals] and they are making maruchan [cup ramen]”.* [P 123S]
	*“Um, you know, like most people or most kids, they love sweet and desserts. We will have, you know, a fruit every once in a while, an ice cream, ice cream bar. Uh, right now with candy season, well, you know, we have some candy, uh, in the pantry. You know, they might take a pizza candy in their lunchbox. Um, you know, so we don’t, you know, something like the last, can we have a treat today? You know, depending, like, we haven’t had one in a couple days for what? Um, but it’s not all the time, you know, not every day”.* [P 118]
	*“I just want him to be active. So, I don’t necessarily call it a workout. I just be like, oh, go, go walk to grandma's house or ride your bike real quick while I’m doing this. Kind of make it seem like it’s a, like I’m letting him have fun instead of just like making it like a chore. I’m making it like a, oh, since I’m doing this, why don’t you go ahead and do that? And he’s like, okay”.* [P 113]
Theme 3: Parents’ Beliefs on Physical Activity and Screen time	*“And almost, almost every weekend we also visit a park or go out to do some outdoor activity together as a family”.* [P 114S]
	*“I think it is [physical activity], it’s important because it also helps her with her growth by, um, being active, I guess. Like, you know, her bones start to stretch and, uh, her muscles and all that. So she’ll have more strength of, um, I guess growing”.* [P 112]
	*“So I like to keep a balance of like him being, ‘cause all the kids are playing video games. All the kids are watching YouTube. And I understand the importance of like, fitting in with your classmates. And also, like, I explained to him like, why we do have parental with you. Even when his friends come over, it’s just like, we’re not doing YouTube. We’re not doing no crazy stuff. Um, and if we do watch YouTube, I’m like right there watching. I’m like, do you think that’s the appropriate? No. And he’ll it. And that’s for all any kid that come over. But screen time is pretty much unlimited, but it’s just supervised because I watch a lot of TV too, so I can’t, I can’t tell him not to watch TV if I watch TV”.* [P 113]
	*“During the weekends I may allow more time [for screens]. … But not during the week, because during the week, they have to do their school routine, reading, writing, practicing. Because they have to have a reading routine”.* [P 128S]
	*“I guess eat healthy is one of them, um, exercising, um, getting enough rest… Um, drinking a lot of, uh, water as well”.* [P 112]
	*“Wow, that’s a touchy subject [screen time]. I already told you that we have here at home I have set schedules for him to, for example, play, play with the Xbox [video game console]. It’s always an hour, an hour and a half on weekends as we all play. So if we expand [continue playing] perhaps a little bit more, he, uh, you know, participates with us when, two hours, three hours playing. Uh, the phone, yes, there have been many, many times that I’ve had to take the phone away from him because uh, he really likes these Minecraft games and Roblox and all these games that are coming out”.* [P 114S]
	*“She comes home from school and tells me Mommy, I’m going to watch TV. But I also try to watch, to be aware of the time she watched TV, for example. I can see that it was already more or less an hour, because sometimes they have no limits. … There they are until you tell them ‘[NAME] you have already watched a lot of TV’ [NAME] stop it, and she stops and turn it off”.* [P 126S]
	*“We have two or three friends that we met in a park. We like the trampolines very much, we always go there as a family or to the lakes when we go fishing”.* [P 114S]
Theme 4: Environmental and Social Factors Affecting Access to Healthy Food and Physical Activity	*“A farmers’ market would be good for fresher fruits if it was also closer because they are far away. I think there may be one closer, but I’ve never been, to be honest”.* [P 115S]
	*“She doesn’t have any friends … Well, yes, that would be beneficial if she had more friends or maybe I could relate more with other people [at a community center]”.* [P 129S]
	*“We live close to, yeah, we live close to the park. So whenever we have time, yeah, they do like to go to the park and either take their bikes or just play with the other friends that we get together. Okay. But yeah, we try to stay there for at least, um, one hour or if we can’t more we’ll do more”.* [P 120]
	*“The parks make it easier. There’s a lot of great parks where I live in the area that I live and I think that makes it, you know, fun for both of us to maintain like some kind of physical activity”.* [P 113]
	*“Yes. We already took him out playing at parks. They get distracted [enjoy themselves] and sometimes meet their friends. Well, I always do it by carrying his water bottle, fruits, some snacks like a cookie”.* [P 122S]
	*“Well my neighborhood is, um, really good place to walk around [and be active]”* [P 112]

## Data Availability

The datasets presented in this article are not readily available because of privacy reasons. Requests to access the datasets should be directed to paolac@unc.edu.

## Supplementary Materials

The following supporting information can be downloaded at: https://www.mdpi.com/article/10.3390/children12070887/s1, Table S1: Questions that guided the Qualitative Interview Guide.
