# Supplementary material for: Factors Influencing Health-Related Practices Among Hispanic Parents: A Formative Study to Inform Childhood Obesity Prevention
Source: Children (Basel). 2025 Jul 5;12(7):887. doi: 10.3390/children12070887 (PMC12293355; doi:10.3390/children12070887)
Supplement: Supplementary file 1 [file children-12-00887-s001.zip › children-3696805-supplementary.pdf]

Supplemental Table S1. Questions that guided the Qualitative Interview Guide

**Icebreaker**	Just to begin, tell me about some foods that remind you of your childhood.
**Overall Parenting Practices**	To start out, tell me about meals in your house.<br>**Probes:** Who prepares the meals in your family? On a school day, what kinds of foods does your child eat? On a weekend? What's a typical breakfast? For lunch, does your child eat a lunch you/they packed or eat a school lunch? What are dinners like at your house? What do you typically eat? What does mealtime look like?<br><br>Next, I'd like to understand how you navigate schedules and routines in your household. Describe what a typical weekday and a typical weekend day looks like for you and your child. Would you walk me through your typical day from when your child wakes up to when they go to sleep?<br>**Probes:** Do you have morning/midday/nighttime routines? What do these look like? What are bedtime routines for your child? When are mealtimes? What kinds of activities does your child do? When does your child engage in these activities?<br><br>I'm interested in learning a bit about your health goals for your child.<br>**Probe**: What does it mean for your child to be healthy?<br><br>From your perspective, what does it mean for your child to be a healthy weight?<br>**Probes:** What are your views on a child being underweight? Overweight?<br><br>What do you consider to be healthy foods? Unhealthy foods?<br>**Probes:** Tell me your thoughts about how you all handle sweets/desserts in your house. What are some ways to encourage healthy eating habits? What can make it hard to encourage healthy eating habits?
**Physical Activity**	Tell me about sports/physical activity in your family.<br>**Probes:** What are some of your child's favorite sports/physical activities? Least favorite? What are some goals you have for physical activity for your child? What do you do together? Do you practice any sport or engage in physical activity that involves you and/or your child? If yes, please describe. If no, is this of interest to you? If so, how would you go about adding that into your week?

	What are things you do that you think have an impact on your child's health and bodyweight?<br>**Probe:** Is there anything else?
**Social and Environmental Factors**	In addition to your child's activities with you or within your family, what other activities does your child engage in? (in school, with friends, in faith-based facilities, in community centers, with family members that do not cohabit, others outside your home).<br><br>What types of things outside of your home make it easier for you and your child to be healthy?
**Prevention and Health-related Recommendations**	Imagine you were talking with a Hispanic/Latino friend or neighbor and sharing your advice on healthy eating and behaviors for children. What would you say to them?<br><br>If we were to develop a program to help Hispanic/Latino families with young children establish healthier eating practices, what are some ideas you have for what should be included in this program? What should we avoid?
**Closing**	Do you have any final thoughts, recommendations, or experiences you would like to share?

Note. Probes were only used if the participant asked for clarification or did not understand the question or statement.
